# Overexpression of *phyA* and *appA* Genes Improves Soil Organic Phosphorus Utilisation and Seed Phytase Activity in *Brassica napus*

**DOI:** 10.1371/journal.pone.0060801

**Published:** 2013-04-03

**Authors:** Yi Wang, Xiangsheng Ye, Guangda Ding, Fangsen Xu

**Affiliations:** 1 National Key Laboratory of Crop Genetic Improvement, and Microelement Research Center, Huazhong Agricultural University, Wuhan, China; 2 College of Resources and Environmental Sciences, Henan Agricultural University, Zhengzhou, China; Nanjing Agricultural Universitym, China

## Abstract

Phytate is the major storage form of organic phosphorus in soils and plant seeds, and phosphorus (P) in this form is unavailable to plants or monogastric animals. In the present study, the phytase genes *phyA* and *appA* were introduced into *Brassica napus* cv Westar with a signal peptide sequence and CaMV 35S promoter, respectively. Three independent transgenic lines, P3 and P11 from *phyA* and a18 from *appA*, were selected. The three transgenic lines exhibited significantly higher exuded phytase activity when compared to wild-type (WT) controls. A quartz sand culture experiment demonstrated that transgenic *Brassica napus* had significantly improved P uptake and plant biomass. A soil culture experiment revealed that seed yields of transgenic lines P11 and a18 increased by 20.9% and 59.9%, respectively, when compared to WT. When phytate was used as the sole P source, P accumulation in seeds increased by 20.6% and 46.9% with respect to WT in P11 and a18, respectively. The P3 line accumulated markedly more P in seeds than WT, while no significant difference was observed in seed yields when phytate was used as the sole P source. Phytase activities in transgenic canola seeds ranged from 1,138 to 1,605 U kg^–1^ seeds, while no phytase activity was detected in WT seeds. Moreover, phytic acid content in P11 and a18 seeds was significantly lower than in WT. These results introduce an opportunity for improvement of soil and seed phytate-P bioavailability through genetic manipulation of oilseed rape, thereby increasing plant production and P nutrition for monogastric animals.

## Introduction

Phosphorus (P) is an essential macroelement required for plant growth. Poor availability of P in soils and consequent P-deficiency are major constraints to crop production globally [1]. Phosphorus is taken up by plants as orthophosphate (P*_i_*). However, in most cultivated soils for agriculture, organic P comprises 30–80% of the total P, and approximately 60–80% of organic P exists in the form of phytate and is not directly available to plants [2], [3]. Thus, improving phytate-P bioavailability is important for plant P nutrition, and for sustainable agricultural development due to the exhausting P ore resources worldwide [4].

Phytase is a type of phosphatase enzyme that catalyses the hydrolysis of phytic acid into inorganic phosphate (P*_i_*) and myo-inositol [5]. Phytases have been most commonly detected and characterised in fungi but have also been found to occur in animals, plants and bacteria [6]. Because phytate in plant seeds is largely indigestible by monogastric animals, much attention has been given to the improvement of phytate-P bioavailability in animal feed by overexpressing phytase genes in plant seeds [4]. The transgenic production of high phytase activity in plant seeds would reduce the costs of producing the enzyme which is currently achieved by fermentation technology [7]. Plant overexpressing either phytase gene *phyA* or *appA* significantly increased phytase activity in wheat grains [8], soybean seeds [9], [10], canola seeds [11], [12] and maize grains [13].

Plants have developed a variety of mechanisms to increase the availability of soil P, including changes in root morphology and architecture, symbiosis with mycorrhizal fungi, improvement of internal phosphatase activity, secretion of organic acids and phosphatases, and up-regulation of high affinity phosphate transporters [3], [1]. Phosphatases are required for the mineralisation of organic P to release phosphate into the soil [14]. Phytases have been identified in roots and root exudates in plants [15], [16], [17]. However, it has been suggested that the enzymatics activity in root exudates is not enough for effective utilisation of organic P [4], [17]. When exogenous phytase was added into the media, phytate became available for plant growth [16], [18], [19]. Therefore, in recent years, more attention has been focused on the potential for overexpressing phytase in plant roots by genetic manipulation for the improvement of organic P use from soil. Overexpression of *phyA* in transgenic *Arabidopsis* with the *Pht1;2* promoter increased extracellular phytase activity and phytate-P use efficiency, and the transgenic plants were able to grow in a medium containing phytate as the sole P source [20], [21]. Transgenic *Trifolium subterraneum* expressing *phyA* showed a 77-fold average increase in exuded phytase activity and took up 1.3- to 3.6-fold more P compared to wild-type plants [22]. Transgenic expression of the phytase gene *MtPHY1* isolated from the model legume *Medicago truncatula* improved phytate-P utilization and plant growth in *Arabidopsis* [23] and white clover [24]. Li *et al*. (2009) expressed the phytase gene *AfPhyA* in soybean roots driven by the *Pky10* promoter and found that the extracellular phytase activity was 6-fold higher than in control roots [25]. Moreover, transgenic cotton overexpressing *phyA* had an improved ability to utilise organic P [26].

These results suggest that overexpression of phytase genes containing a secretion signal improve phytate-P utilisation. *Brassica napus* is an important oilseed crop worldwide, but it is sensitive to P deficiency [27]. Moreover, the widespread use of oilseed meal in livestock feed and the inability to utilise phytate-P can lead to serious environmental consequences [1]. In the present study, phytase genes *phyA* and *appA* from *Aspergillus niger* and *Escherichia coli*, respectively, were used to generate transgenic *Brassica napus* plants that could secrete phytase as an extracellular enzyme. The results showed that transgenic plants not only significantly increased their capacity of P acquisition from phytate, but they also produced stable recombinant phytase in seeds. This study could have significant implications for the improvement of phytate-P bioavailability in soil and seeds, for increased plant production and for P-nutrition for monogastric animals from a single transgenic plant.

## Results

### Generation of transgenic lines overexpressing phytase genes

**Figure 1:**
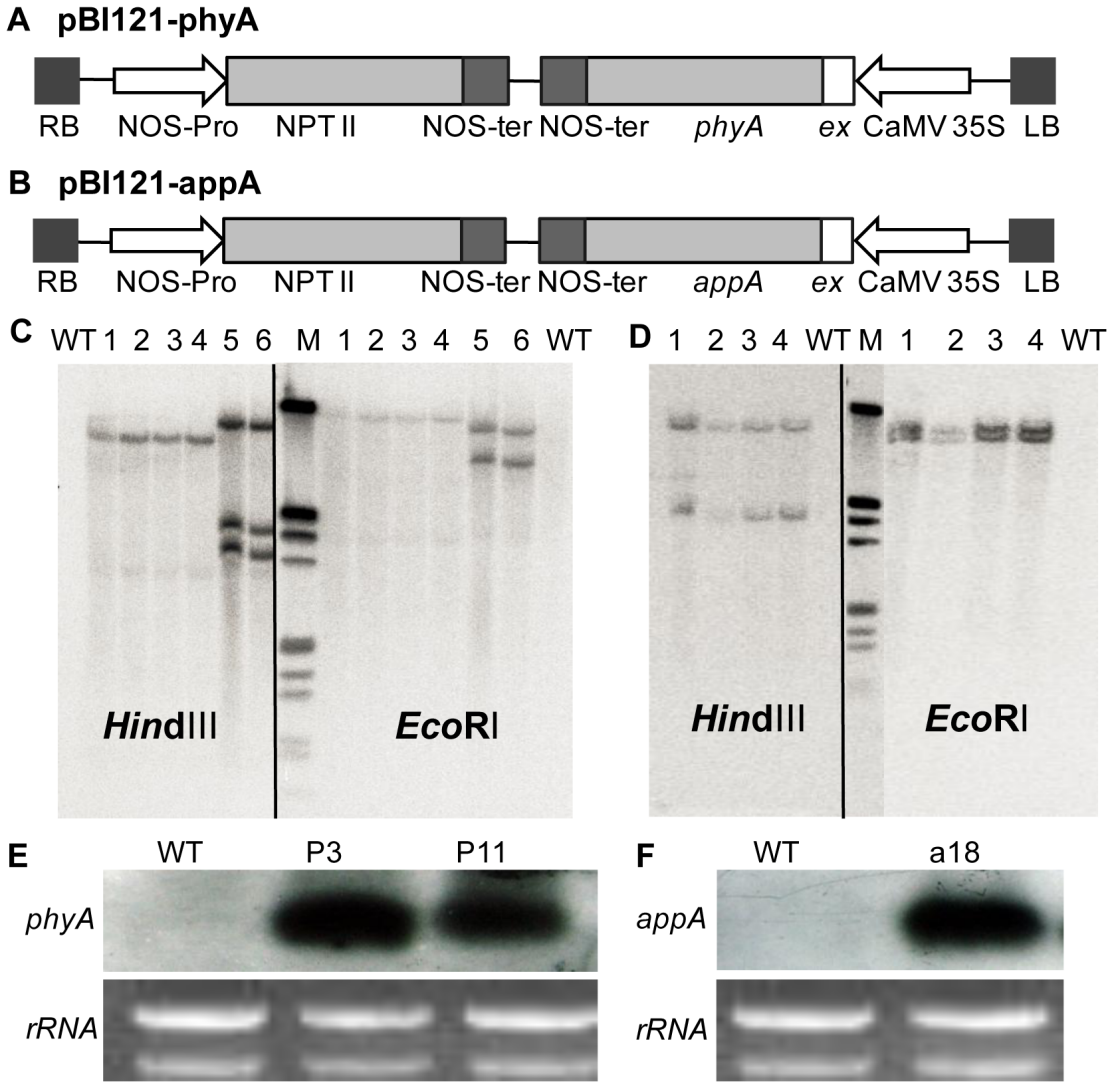
Construction of transgenic plants overexpressing *A. niger phyA* or *E. coli appA*. **A** and **B**: Schematic representation of binary vector pBI121-phyA (**A**) and pBI121-appA (**B**) for *B. napus* plant transformation. Phytase genes (*A. niger phyA* and *E. coli appA*) under the control of the CaMV 35S promoter and nos-terminator were modified for extracellular secretion by inclusion of an extracellular targeting sequence from the carrot extensin (*ex*) gene. The construct has a selectable marker for the *NPT*?? gene. **C** and **D**: Southern blotting analysis of *ex::phyA* (**C**) and *ex::appA* (**D**) transgenic lines, using *phyA* and *appA* probes, respectively. Genomic DNA was digested by *Hind*III and *EcoR*I. **C**, M, Marker; WT, wild-type canola; 1–4,T_3_ transgenic plants of P3 line; 5–6, T_3_ transgenic plants of P11 line; **D**, 1–4, T_3_ transgenic plants of a18 line. **E** and **F**: Northern blotting analysis of *phyA* (**E**) and *appA* (**F**) expression in roots of transgenic lines using *phyA* and *appA* probe, respectively.

The plasmids pBI121-phyA and pBI121-appA (Fig. 1A, B) were used for the transformation of *B. napus* cv Westar. A total of 28 independent transformation events were obtained, 22 with pBI121-appA and 6 with pBI121-phyA. First, the presence of *A. niger phyA* or *E. coli appA* in the transformed lines was confirmed by genomic PCR analysis (data not shown). The positive T_0_ plants were self-crossed to ultimately obtain T_3_ transgenic lines for further analysis. Three canola transgenic lines, P3 and P11 with *phyA* and a18 with *appA*, were selected based on their high phytase activity in root extracts and lack of segregation with kanamycin-resistant screening. Southern blotting of the three T_3_ transgenic lines demonstrated that the exogenous *phyA* and *appA* were correctly integrated into the genome of transgenic canola plants (Fig. 1C, D). P3 and P11 had one and three copies of *phyA* gene, respectively (Fig. 1C), and the a18 line had two copies of the *appA* gene (Fig. 1D). The mRNA expression of *A. niger phyA* and *E. coli appA* in the roots of the three transgenic lines was analysed by northern blotting (Fig. 1E, F). Each transgenic line showed high accumulation of either *phyA* or *appA* transcripts. By contrast, no hybridisation bands corresponding to *A. niger phyA* or *E. coli appA* were detected in wild-type (WT) plants.

### Phytase activity in transgenic *Brassica napus*

**Figure 2:**
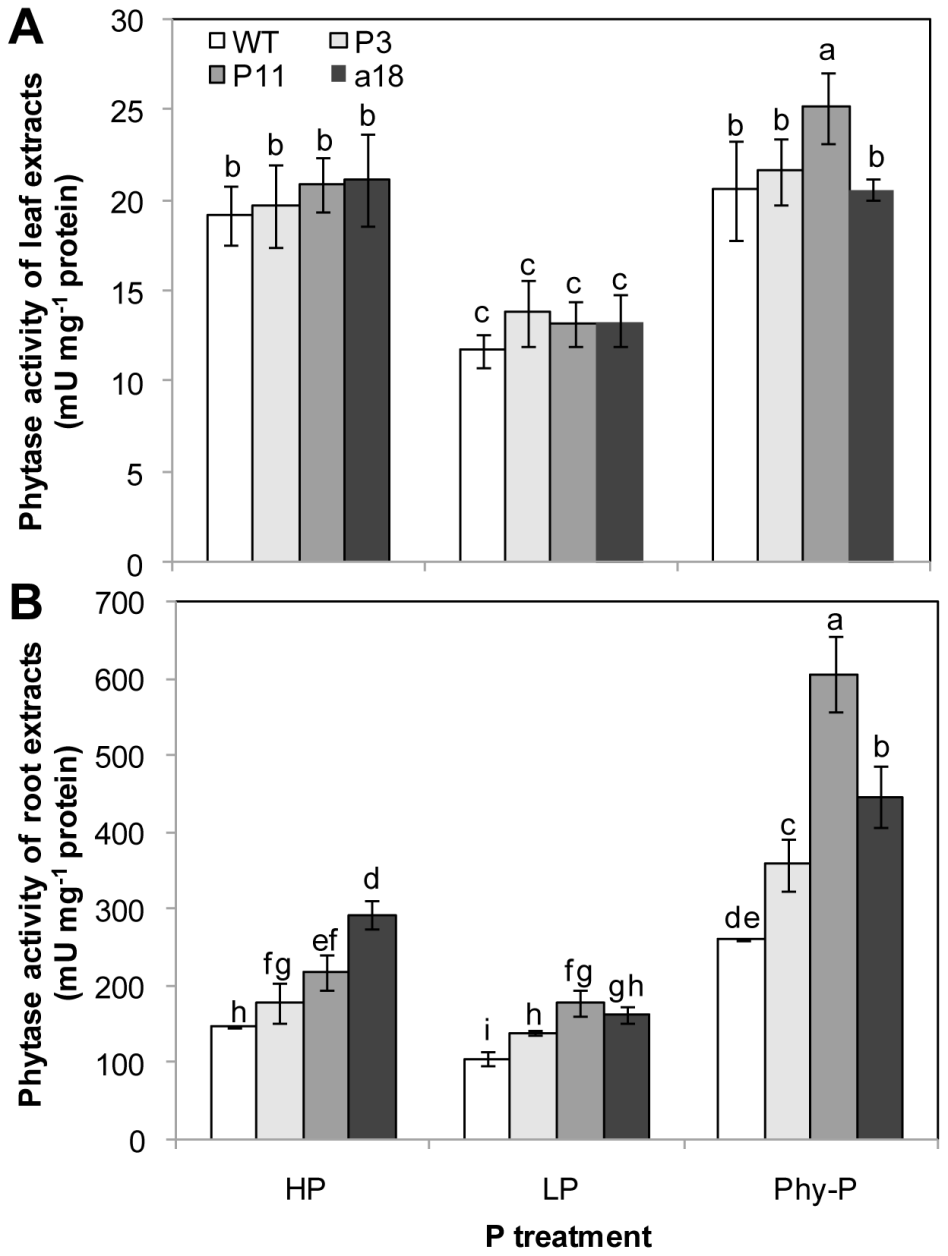
Phytase activity in leaf (A) and root (B) extracts of transgenic lines when grown in different P conditions. HP: plants were grown in high P (250 µM KH_2_PO_4_) nutrient solution for 18 days; LP: plants were grown in low P (5 µΜ KH_2_PO_4_) nutrient solution for 18 days; Phy-P: plants were grown in 250 µM P in the form of phytate for 18 days. Each column is the mean of four replicates with SD. Different letters represent significant differences at the *p<*0.05 level.

There was no significant difference in phytase activity in leaf extracts between transgenic lines and WT with the exception of P11 in Phy-P treatment (Fig. 2A). Phytase activities were higher in *ex::phyA* and *ex::appA* root extracts than in WT plants regardless of P availability (Fig. 2B). When phytate was added as a P source, phytase activity significantly increased compared to other P treatments, and the activity in the P11 line was nearly two-fold higher than in WT.

**Figure 3:**
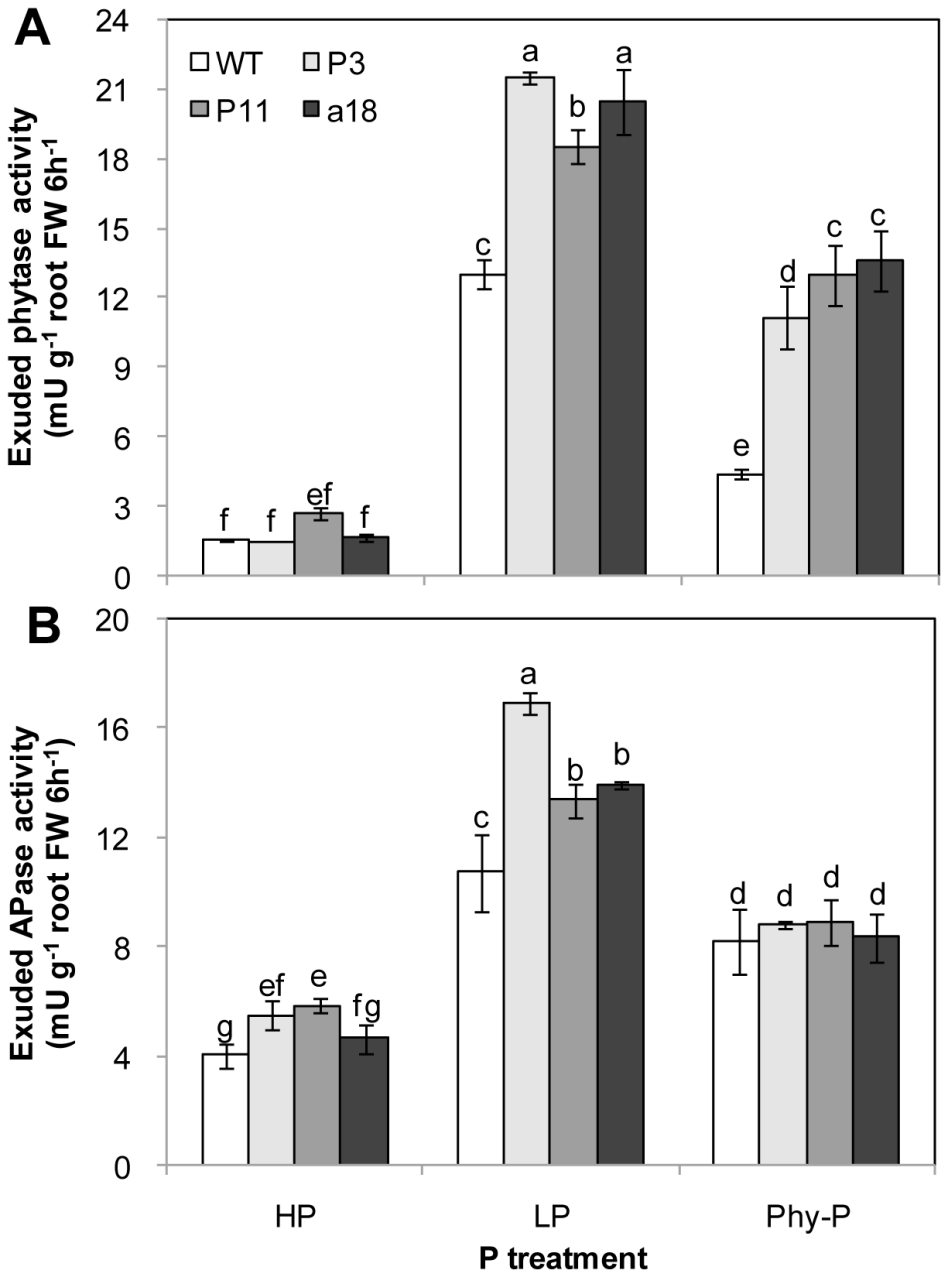
Phytase activity (A) and acid phosphatase activity (B) exuded from roots of transgenic lines expressing *ex::phyA/appA* and WT. Plants were grown in different P treatment solutions. Each column is the mean of four replicates with SD. Different letters represent significant differences at the *p<*0.05 level.

Exuded phytase activity was markedly higher in transgenic lines than in WT when grown in P-deficient conditions (Fig. 3A). With Phy-P treatment, exuded phytase activities were increased approximately 3-fold in *ex::phyA* and *ex::appA* lines when compared to WT plants. The exuded phytase activity was very low at high P (HP) supply, and this was not significantly different between transgenic lines and WT (Fig. 3A). In low phosphorus (LP) treatment, exuded acid phosphatase (APase) activity was significantly higher in transgenic lines than in WT (Fig. 3B). However, when grown in Phy-P, no significant difference in exuded APase activity was observed between transgenic lines and WT (Fig. 3B).

### Growth and P nutrition of transgenic lines in sand culture experiment

**Figure 4:**
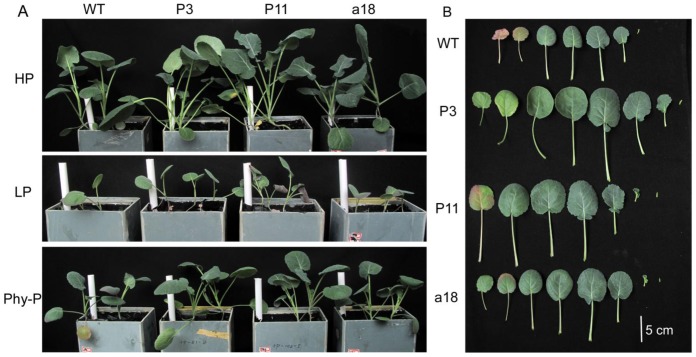
Growth response of transgenic plants and WT plants in sand culture. **A**: Phenotype of transgenic lines (P3, P11 and a18) and WT plants grown for 60 d in quartz sand culture with different P sources. **B:** Leaf number and size of transgenic lines and WT plants when grown in quartz sand with phytate as the sole P source. The leaves arranged from left to right ranged from the oldest to youngest according to the growth order in the plant.

A quartz sand culture experiment was performed to evaluate the response of transgenic plants to the supply of phytate-P in the growth medium. Vegetative growth phenotypes indicated that the three transgenic lines had larger shoots than WT in the three treatments (Fig. 4A). When grown in Phy-P-treated sand, transgenic lines showed much larger leaf sizes and more leaves compared to WT plants (Fig. 4B). Moreover, WT plants exhibited serious P-deficiency symptoms in their old leaves, while no evident P-deficiency was observed in P3 leaves with Phy-P treatment (Fig. 4B).

When phytate was used as the sole P source, the three transgenic lines P3, P11 and a18 had more P accumulation in the shots than WT by 70.8%, 36.8% and 19.6%, respectively (Table 1). Moreover, shoot biomass increased from 14.7% to 36.8% in the transgenic lines compared to WT. In HP treatment, P accumulation in transgenic shoots increased from 41.2% to 70.9% and shoot biomass increased from 28.2% to 53.4% compared to WT. And in LP treatment, the P3 line exhibited a 44.4% increase in shoot biomass and a 167% increase in total P content compared to WT (Table 1).

**Table 1:** Shoot biomass, P concentration and P accumulation of transgenic lines and WT grown in quartz sand.

P treatment	Line	Dry weight	P concentration	P accumulation
		g plant^–1^	%	mg plant^–1^
HP	WT	1.03±0.07	0.42±0.04	4.30±0.21
	P3	1.32±0.16*	0.47±0.04	6.07±0.81**
	P11	1.58±0.21**	0.49±0.05	7.22±0.88**
	a18	1.39±0.23*	0.57±0.03*	7.35±1.23**
LP	WT	0.09±0.01	0.14±0.01	0.12±0.01
	P3	0.13±0.01*	0.24±0.01*	0.32±0.03**
	P11	0.13±0.02*	0.17±0.02	0.18±0.01*
	a18	0.10±0.02	0.16±0.00	0.18±0.01*
Phy-P	WT	0.68±0.04	0.31±0.04	2.09±0.43
	P3	0.78±0.08*	0.43±0.04*	3.57±0.34**
	P11	0.93±0.05**	0.31±0.03	2.86±0.34*
	a18	0.93±0.08**	0.27±0.01	2.50±0.21*

Values represent means ± SD of four replicates. A significant difference (*, ** at *P*<0.05 and *P*<0.01, respectively) between WT and transgenic lines in the same P treatment was calculated by LSD test.

### Seed yields and P accumulation in transgenic lines

To detect the transgenic plant growth in P-deficient soil during the entire growth period, we conducted a pot culture experiment. Under Phy-P treatment, transgenic lines P11 and a18 had significant increases in seed yields of 20.9% and 59.9%, respectively, compared to WT (Table 2). In LP treatment, only the a18 line improved seed yields, with a 46.1% increase with respect to WT. Seed yields from P11 and a18 lines with HP treatment were 62.5% and 55.5% higher than in WT, respectively (Table 2). However, no significant difference was observed between the seed yields of the P3 line and WT in the three P treatment soils (Table 2). In HP treatment, P accumulation in seeds of transgenic lines P11 and a18 increased by 41.8% and 59.9%, respectively, compared with WT. Transgenic line P3, P11 and a18 respectively accumulated 11.5%, 20.6% and 46.9% more P in seeds than WT when plants were grown in P-deficient soil to which only phytate was applied (Table 2). However, in LP treatment, only the a18 line accumulated more P in seeds than WT (Table 2). Overexpression of *phyA* and *appA* in *B. napus* can improve the seed yields of transgenic plants and accumulated more P in seeds when phytate was the sole P source.

**Table 2:** Effects of different P sources on seed yield, P concentration, P accumulation, phytase activity and phytic acid content of WT and transgenic lines grown in P deficient soil.

P treatment	Line	Seed yield	P concentration	P accumulation	phytase activity	phytic acid content
		g plant^–1^	%	mg plant^–1^	U kg^–1^ seeds	mg g^–1^ seeds
HP	WT	4.99±0.60	0.81±0.01	40.55±4.31	n.d.	29.57±1.08
	P3	4.66±0.08	0.83±0.06	35.81±2.44	938.4±32.8	30.20±0.86
	P11	8.11±0.71**	0.71±0.04*	57.51±7.91**	871.9±34.1	19.20±2.81**
	a18	7.76±0.60**	0.82±0.04	64.85±6.74**	1251.6±35.5	19.89±3.12**
LP	WT	1.80±0.12	0.47±0.07	8.84±1.07	n.d.	23.13±0.82
	P3	1.08±0.19	0.45±0.07	4.63±0.06*	620.1±50.7	20.44±1.33*
	P11	1.37±0.26	0.42±0.04	5.25±0.68*	672.5±38.4	11.33±1.51**
	a18	2.63±0.25*	0.35±0.03*	9.31±1.66*	344.7±13.5	16.92±1.25**
Phytate-P	WT	4.32±0.28	0.65±0.03	27.01±1.59	n.d.	24.24±3.54
	P3	3.79±0.16	0.80±0.03*	30.09±0.69*	981.8±79.5	24.44±2.39
	P11	5.21±0.17*	0.62±0.04	32.57±2.97*	1116.1±88.9	13.04±1.38**
	a18	6.91±0.48**	0.62±0.07	39.69±3.97**	707.2±42	16.80±1.92**

Values represent means ± SD of four replicates. A significant difference (*, ** at *P*<0.05 and *P<*0.01, respectively) between the WT and transgenic lines in the same P treatment was calculated by LSD test. HP treatment: 200 mg P_2_O_5_ kg^–1^ soil with KH_2_PO_4_; LP treatment: 20 mg P_2_O_5_ kg^–1^ soil with KH_2_PO_4_; Phy-P treatment: 200 mg P_2_O_5_ kg^–1^ soil with phytate. n.d., means not detectable.

### Phytase activity and phytic acid content in seeds

**Figure 5:**
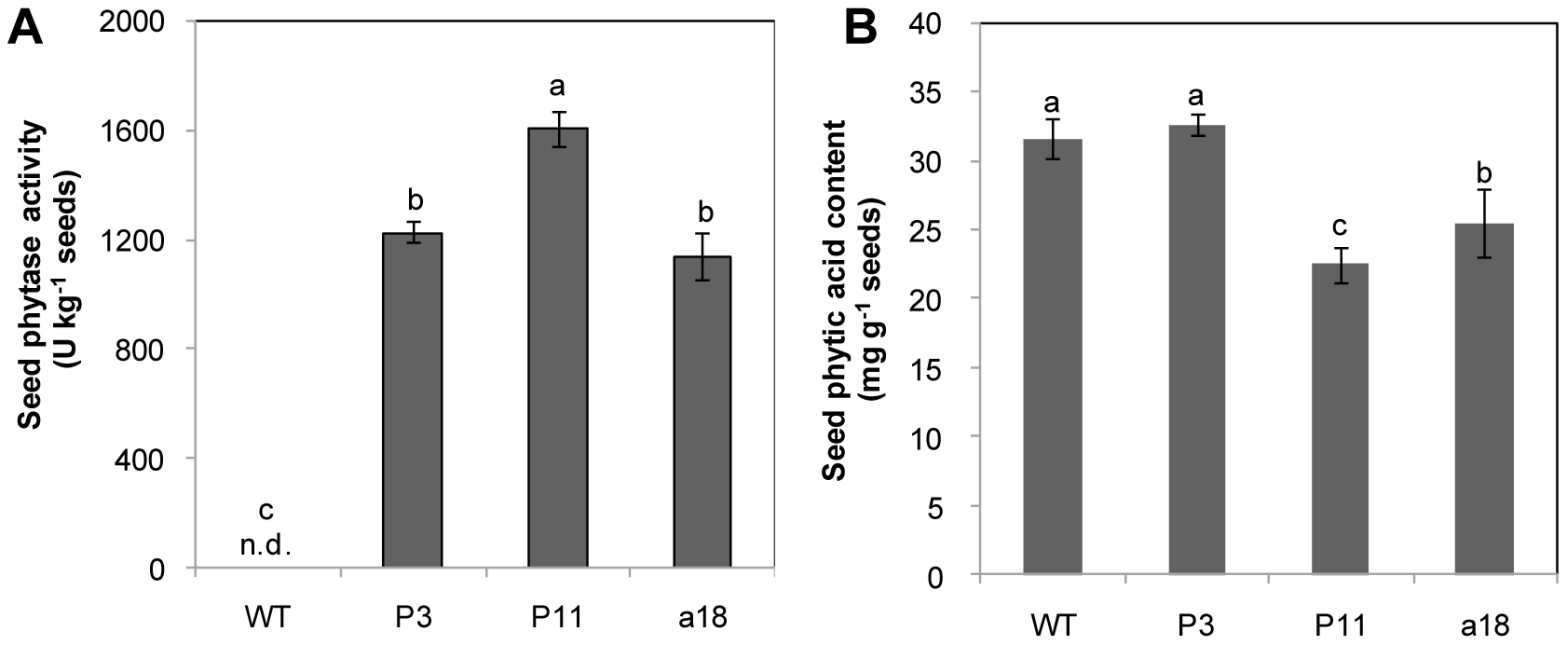
Phytase activity (A) and phytic acid content (B) in seeds of transgenic T_3_ lines and WT. Each column is the mean of four replicates with SD. Different letters represent significant differences at the *p<*0.05 level. The seeds were harvested from the field by self-cross. n.d., means not detectable.

T_3_ seeds harvested from the field by self-cross were assayed for phytase activities and phytic acid contents. Phytase activities for P3, P11 and a18 were 1,225, 1,605 and 1,138 U kg^–1^ seeds, respectively (Fig. 5A). However, no phytase activity was detected in WT seeds. The phytic acid content in WT seeds was approximately 31.6 mg g^–1^ seeds, and it was reduced to 22.5 and 25.5 mg g^–1^ seeds in P11 and a18 transgenic seeds, respectively (Fig. 5B). However, no significant difference in phytic acid content was observed between the P3 line and WT seeds.

The results of the soil pot culture experiment indicated that phytase activity in the three transgenic lines ranged from 344.7 to 1251.6 U kg^–1^ seeds when grown in the presence of HP, LP and Phy-P treatments (Table 2). However, no phytase activity was detected in WT seeds. The phytic acid content in WT seeds was approxiamtely 30 mg g^–1^ seed, while it was reduced to 19.2 and 19.9 mg g^–1^, respectively, in P11 and a18 transgenic seeds with HP treatment (Table 2). The phytic acid content in P11 and a18 seeds was reduced by 46.2% and 30.7%, respectively, compared to WT seeds when phytate was the sole P source (Table 2). However, the phytic acid content of P3 seeds was not reduced compared with WT in either of the two treatments, which was consistent with the results in Figure 8B. When grown in LP-treated soil, phytic acid content was significantly reduced in the three transgenic lines compared to WT.

## Discussion

In total, 30% to 80% of the total P in agricultural soils exists as organic phosphate, and up to 80% of organic phosphate is in the form of phytate-P [2], [28]. Because phytate-P is not directly available to plants, low P availability becomes one of the limiting factors for plant growth. Phytases were identified in plant roots and root exudates [15], [16]; however, the activity of these enzymes in roots is inadequate for efficient utilisation of organic P from soil [16], [17]. In the present study, the exudate phytase activities were very low in WT plants when grown in HP treatment. However, the secreted phytase activity increased in WT plants in P-stressed conditions (LP and Phy-P) (Fig. 3A). This suggested that P starvation induced canola to exude phytase into the rhizosphere depending on P availability. Hayes et al. (2000) and Richardson et al. (2000) showed that a low intrinsic level of phytase activity in plant roots exudates is a critical factor that limits the ability of plants to obtain P from phytate [16], [17]. Idriss et al. (2002) reported that the presence of purified FZB45 phytase promoted the growth of maize seedlings when phytate was the sole P source [18]. In the present study significant increase in extracellular phytase activity was observed in transgenic lines expressing *ex::phyA* or *ex::appA* compared to WT in P deficient conditions, which is consistent with Richardson et al. (2001) and George et al. (2004) [20], [22]. No significant difference in extracellular phytase activity was observed between the transgenic lines and WT plants in HP treatment (Fig. 3A), even though the exogenous *ex::phyA* and *ex::appA* gene were highly expressed (Fig. 1E, F). However, the difference was significant under P-stressed conditions (Fig. 3A). Phytase activity was much higher in root extracts from the transgenic lines than from WT (Fig. 2B), suggesting that the exudation of phytase into the rhizosphere is induced by P limitation. The phytase secretion mechanism induced by P deficiency together with the extracellular targeted signal peptide sequence may have contributed to phytase exudation in the transgenic lines.

Enhanced exudation of phytase by overexpressing *ex::phyA* or *ex::appA* in transgenic *B. napus* conferred the ability to acquire P from phytate. All *ex::phyA/appA* lines exhibited increased P uptake and shoot biomass production in comparison with WT when grown in quartz sand supplied with phytate as the only source of P (Table 1). The increase in shoot biomass was comparable to the 30% reported by Zimmermann et al. (2003) in transgenic potato [29], the 23% increase observed by Mudge et al. (2003) in *Arabidopsis* [21] and the 27% increase observed by George et al. (2004) in *Trifolium subterraneum* L. [22]. The observed increase in total P content of shoots in *ex::phyA/appA B. napus* corresponds well to the 40% increase in P uptake observed in transgenic potato [29], the 3-fold increase observed in transgenic *Arabidopsis* [21] and the 1.3- to 3.6-fold increase observed in transgenic *T. subterraneum* [22]. Moreover, the dry weight of the transgenic plants increased from 28.2% to 53.4% and total P content increased from 50.0% to 167% compared to WT when grown in HP and LP treatments in sand culture.

In HP and Phy-P treatment soils, seed yield and phytic acid content in P11 were significantly higher than in WT plants. However, no difference was observed between P3 and WT (Table 2). This might de due to the difference in copy number of the *phyA* gene in the two transgenic lines. Hong et al. (2004) found that transgenic lines 8FN-8 and 8AN-14, which contain three and four copies of their transgenes, respectively, showed the highest phytase activity compared to other transgenic lines. They suggested that multiple copies of transgenes could, but may not necessarily, contribute to higher expression levels of phytase in germinated rice seeds [30]. The bacterial phytases have broader pH optima and higher specific activities compared to the fungal phytase [31], which may partly explain the difference in seed yield, phytase activity and phytic acid content between *phyA* lines and *appA* line. However, this speculation needs to be further verified by using abundant independent transgenic lines with different copies of the *phyA* and *appA* genes.

Phytate is the main storage form of P in plant seeds, which are a major component of animal feed [32]. Phytic acid is largely indigestible by monogastric animals because they have no or limited phytase activity in their digestive tract [4]. Thus, in addition to improving P uptake from soil, transgenic expression of phytase in plant seeds offers prospects for improving phytate-P bioavailability in monogastric animals. Previous studies indicated that the overexpression of phytase genes in soybean [9], [10], maize [13], canola [11], [12], rice [30] and wheat [7], [8] seeds improved phosphorus availability and reduced the impact of animal production on the environment. In the present study, in addition to improving phosphorus uptake from phytate, overexpression of *ex::phyA* or *ex::appA* in canola produced stable recombinant phytase and significantly reduced phytic acid content in transgenic seeds. Phytase activities in transgenic seeds were 1,138 to 1,605 U kg^–1^ seeds (Fig. 5A), which were close to the 2,000 U kg^–1^ seeds observed by Chen *et al*. (2008) in transgenic maize [13]. Moreover, nearly no phytase activity was detected in WT canola seeds. The role of the signal sequence in carrot extensin (*ex*) is to transport extensins through the cell membrane to reach the cell wall [33], [34]. In transgenic plants expressing a phytase genes with the *ex* signal peptide sequence, researchers have historically only focused on the extracellular phytase activity and soil phytate-P uptake but have not noted the changes in seed phytase activity [20], [21], [22], [35]. Our results demonstrate that the overexpression of *ex::phyA* or *ex::appA* in *B. napus* not only increased extracellular phytase activity but also accumulated phytase in seeds. This result indicated that expression of the phytase gene with the signal peptide sequence from *ex* gene may improve both the P uptake in roots and the bioavailability of phytate-P in seeds in one transgenic plant.

In conclusions, transgenic *B. napus* plants expressing *ex::phyA* or *ex::appA* significantly improved P uptake, plant biomass and seed yields when phytate was supplied as the sole P source. Moreover, transgenic seeds showed high phytase activity and significantly reduced phytic acid content. For the first time in one transgenic plant, these results indicate that the transgenic approach demonstrates great potential for improving plant P acquisition and animal feed supplement.

## Materials and Methods

### Plant transformation

Expression cassettes of *Aspergillus niger* phytase (*phyA*, Accession no. M94550.1) and *Escherichia coli* phytase (*appA*, Accession no. M58708.1) including a signal peptide sequence from the carrot extensin gene were excised from the 35S-phyA and 35S-appA plasmids (provided by Profs. Ping Wu and Huixia Shou, Zhejiang University, China) with *Hind*III and *EcoR*I, respectively. It was then subcloned into the pBI121 binary vector from which the *GUS* expression cassette had been previously removed with *Hind*III and *EcoR*I resulting in the transformation vectors pBI121*-*phyA and pBI121-appA. The two microbial phytase genes, under the control of the CaMV 35S promoter and nos terminator, were modified for extracellular secretion by inclusion of a signal peptide sequence (Fig. 1A, B). The pBI121-phyA and appA plasmids were transformed into *B. napus* cv Westar plants by an *Agrobacterium tumefaciens* (LBA4404) mediated method described by Wang *et al*. (2012) [36]. All tissue culture materials were maintained in a controlled growth chamber (24°C/16 h of light, 23°C/8 h of dark, and a light intensity of 80 µmol m^–2^ s^–1^).

### Molecular identification of transformants

For Southern blotting analyses, genomic DNA was isolated from leaves using the cetyltrimethyl ammonium bromide extraction method [37]. Thirty micrograms of genomic DNA digested with *Eco*RI or *Hin*dIII was separated on a 0.8% agarose gel and transferred to nylon membranes, which were hybridised with probes from *A. niger phyA* and *E. coli appA* α-P^32^ dCTP-labelled PCR products. The 441 bp PCR product was amplified with *A. niger phyA* specific primers 5′-ACAGACACGAAGTGACCTACC-3′ and 5′-CCAAGCAGACGAGAATCC-3′. Specific primers 5′-GAACGTGACTGACGCGATCC-3′ and 5′-ACGCCAGCGTTCAAACACC-3′ were used to amplify 565 bp of *E. coli appA*.

For northern blotting analysis, total RNA was extracted from roots with TRIzol reagent (Invitrogen, CA, USA) according to the manufacturer’s instruction, and separated by electrophoresis on agarose formaldehyde denaturing gels. The separated RNA was then transferred to nylon membranes, which were hybridised with the probes from the α-P^32^ dCTP-labelled PCR products of *A. niger phyA* and *Ecoli appA*.

### Solution culture experiment

Seeds from WT and transgenic canola lines P3, P11 and a18 were surface-sterilised for 1 min using 10% (*w*/*v*) sodium hypochlorite and washed four times in deionised water. The surface-sterilised seeds were then germinated on moistened gauze that was fixed to a black plastic tray filled with deionised water at 25°C. After 5 to 6 days, uniformly sized seedlings were transferred to a 10 L black plastic tray (53.0 cm × 34.0 cm×7.5 cm) filled with Hoagland’s nutrient solution modified to one-fourth strength [38]. After 5 days, the nutrient solution was replaced with half strength nutrient solution, which was replaced again with full strength nutrient solution after another 5 days. The composition of the full strength nutrient solution was 5.04 mM KNO_3_, 0.25 mM KH_2_PO_4_, 1.99 mM MgSO_4_·7H_2_O, 5.0 mM Ca(NO_3_)_2_·4H_2_O, 0.32 µM CuSO_4_·5H_2_O, 0.77 µM ZnSO_4_·7H_2_O, 9.14 µM MnCl_2_·4H_2_O, 0.37 µM NaMoO_4_·2H_2_O, 46 µM H_3_BO_3_ and 0.07 mM EDTA-Fe. The pH of the nutrient solution was controlled to 5.5±0.2 with 1 M NaOH or HCl every 2 days. The seedlings were grown in an illuminated culture room at a cycle of 16 h/24°C day and 8 h/22°C night and a light intensity of 300–320 µmol m^–2^ s^–1^ with a relative humidity of 65–80%.

In the low P stress experiment, three P levels were designed with four replicates per level. Both WT and transgenic canola seedlings were grown for 18 days in high P (HP) and low P (LP) nutrient solutions containing 250 µM and 5 µM P, respectively. For Phy-P treatment, the seedlings were grown in 250 µM P (C_6_H_6_O_24_P_6_Na_12_, phytate, P3168, Sigma) for 18 days. In the LP and Phy-P solutions, KH_2_PO_4_ was replaced by K_2_SO_4_.

Root exudate collection was performed according to the method described by George et al. (2004) and Richardson et al. (2001) with some modifications [22], [20]. The seedlings (2 seedlings per biological replicate) from different P treatments were transferred to 15 mL of 15 mM MES (2-(*N*-morpholino)-ethanesulfonic acid monohydrate) buffer (pH 5.5) containing 0.5 mM CaCl_2_ (MES/Ca buffer) for 6 h. The fresh weight of shoot and root material was measured and frozen in liquid nitrogen. The exudate was filled to 15 mL with the MES/Ca buffer and stored at 4 °C prior to the enzyme assays. All treatments contained four biological replicates and two plants in each biological replicate.

For the enzyme extraction in leaves and roots, approximately 0.4 g of leaves or roots was ground in liquid nitrogen and homogenised with 2 mL MES/Ca buffer containing 1 mM EDTA. Extracts were then centrifuged (10 min, 12,000 g) and subsequently used to determine phytase activities. All treatments contained four biological replicates and two plants in each biological replicate.

### Determination of enzyme activities

Phytase and APase activities were determined using C_6_H_6_O2_4_P_6_Na_12_ (phytate, P3168, Sigma) and *p*-nitrophenyl phosphate (*p*NPP), respectively. The activities were expressed in either mU g^–1^ root FW or mU mg^–1^ protein, where 1 U is defined as the release of 1 mmol of P*_i_* (phytase) or *p*-nitrophenol (*p*NP) (APase) min^–1^ under the assay conditions described.

Phytase activity in extracts was measured as described by Richardson et al. (2000) with some modifications [17]. The phytase assay was conducted by incubating 500 µL extract in a total volume of 1 mL MES/Ca buffer (pH 5.5) at 37°C for 60 min in the presence of 1 mM C_6_H_6_O2_4_P_6_Na_12_ (pH 5.5). The reaction was terminated by the addition of an equal volume of 10% TCA (trichloroacetic acid). Phytase activity was calculated from the release of P*_i_* in centrifuged samples (12,000 g, 10 min) and was determined using the molybdate-blue assay [39]. The protein content of root extracts was measured using bovine serum albumin standards, according to Bradford (1976) [40].

Extracellular phytase and APase activity analysis were performed as described by Richardson et al. (2000) with some modifications [17]. Root exudates (950 µL) were combined with 50 µL substrates (final concentration 1 mM for phytate and 0.5 mM for *p*NPP) and incubated at 37°C for 60 min (phytase) or 30°C for 30 min (APase). Reactions were terminated with an equal volume of either 10% TCA (phytase) or 0.25 M NaOH (APase) at the end of the incubation. APase activity was calculated from the release of *p*NP, as determined spectrophotometrically at 405 nm relative to standard solutions. Phytase activity was calculated as described above.

### Quartz sand culture experiment

For the quartz sand culture experiment, seeds of WT and transgenic lines were surface-sterilised and planted into a plastic box (17 cm×8 cm×17 cm) filled with sterilised quartz sand. For inhibiting moss growth, a black plastic membrane was used to cover the surface of the quartz sand. The plants were fertilised with the modified Hoagland’s nutrient solution described above with different P concentrations. In high P (HP) and low P (LP) treatments, nutrient solutions contained 250 µM and 5 µM P (KH_2_PO_4_), respectively. In Phy-P treatments, phytate (C_6_H_6_O_24_P_6_Na_12_, P3168, Sigma) was added as the sole P source and the P concentration was 250 µM. Each treatment contained four biological replicates with two plants in each replicate. Sixty days after germination, the plants were harvested for shoot biomass and P content determination. The total P was determined according to the vanadomolybdate method described by Westerman (1990) after digesting the samples in concentrated H_2_SO_4_-HClO_4_ using a digestion block system [41].

### Soil culture experiment

Seeds of WT and transgenic lines were surface sterilised and then sowed into a pot filled with 7.0 kg of soil from the Huazhong Agricultural University campus (Hubei province, China, 30.47°S 114.35°E). Soil characteristics were as follows: pH 6.19, total nitrogen 0.85 g kg^–1^, total phosphate 0.21 g kg^–1^, Olsen-P 2.68 mg kg^–1^ and organic matter 5.14 g kg^–1^. Three treatments were employed including 20 mg P_2_O_5_ kg^–1^ soil (LP), 200 mg P_2_O_5_ kg^–1^ soil (HP) (added as KH_2_PO_4_) and 200 mg P_2_O_5_ kg^–1^ soil added as C_6_H_6_O2_4_P_6_Na_12_ (phytate, P3168, Sigma). Nitrogen (N) and potassium (K) fertilisers were mixed with the soil before planting at a concentration of 200 mg N and K_2_O kg^−1^ soil in the form of (NH_4_)_2_SO_4_ and KCl. Other nutrients were supplied as a nutrient solution containing 25 mg kg^–1^ MgSO_4_·7H_2_O, 2.86 mg kg^–1^ H_3_BO_3_, 1.81 mg kg^–1^ MnCl_2_·4H_2_O, 0.22 mg kg^–1^ ZnSO_4_·7H_2_O, 0.08 mg kg^–1^ CuSO_4_·5H_2_O and 0.02 mg kg^–1^ (NH_4_)_6_MO_7_O_2_·4H_2_O. Eight months after germination, plants were harvested for yield evaluation, seed phytase activity and phytic acid content analysis. Total P in seeds was determined as described above. Each treatment contained four biological replicates and two plants in each replicate.

### Determination of phytase activity and phytic acid content in seeds

Seeds were powdered in liquid nitrogen, and 100 mg of sample was extracted in 2 mL of enzyme buffer (0.1 M sodium acetate, pH 5.5, 1 mM CaCl_2_, and 0.1 g L^–1^ Tween 20) by thorough vortex mixing, followed by incubation on ice for 10 min. Samples were centrifuged for 5 min at 15,000 g, 4°C [42]. The supernatants were transferred into fresh tubes and used for phytase activity measurements with the method described by Wyss et al. (1999) [5]. Briefly, 200 µL supernatant was mixed with 700 µL of 0.1 M sodium acetate buffer and 100 µL of 1 mM phytate and incubated at 37°C for 30 min. The reaction was stopped by adding 1 mL of 10% (v/v) TCA. As a control, TCA was added to the supernatant, followed by the phytate substrate and incubated in the same conditions. Phytase activity was calculated as described above.

Phytic acid content was assayed according to the methods from Latta and Eskin (1980) and Kwanyuen and Burton (2005) with some modifications [43], [44]. Samples of 100 mg were extracted with 2 mL of 0.4 M HCl–15% TCA at 23°C for 3 h. After centrifugation at 3,000 g for 15 min, 20 µL of the supernatant was mixed with 280 µL of 36.3 mM NaOH and 200 µL of 0.03% (w/v) FeCl_3_·6H_2_O–0.3% sulfosalicylic acid. The mixture was centrifuged at 2,000 g for 10 min. The absorbance of the supernatant at 500 nm was measured using an Ultrospec 1100 *pro* spectrophotometer (Biochrom Ltd, England).

### Statistical analysis

The data were analysed statistically using the SAS program (Statistical Analysis Systems Institute, version 8.1).
